# Utility of quantitative measurement of *T*_2_ using restriction spectrum imaging for detection of clinically significant prostate cancer

**DOI:** 10.1038/s41598-024-82742-8

**Published:** 2024-12-28

**Authors:** Mariluz Rojo Domingo, Christopher C. Conlin, Roshan Karunamuni, Courtney Ollison, Madison T. Baxter, Karoline Kallis, Deondre D. Do, Yuze Song, Joshua Kuperman, Ahmed S. Shabaik, Michael E. Hahn, Paul M. Murphy, Rebecca Rakow-Penner, Anders M. Dale, Tyler M. Seibert

**Affiliations:** 1 Department of Bioengineering, University of California San Diego Jacobs School of Engineering, La Jolla, CA USA; 2 Department of Radiation Medicine and Applied Sciences, University of California San Diego School of Medicine, La Jolla, CA USA; 3 Department of Radiology, University of California San Diego School of Medicine, La Jolla, CA USA; 4 Department of Electrical and Computer Engineering, University of California San Diego Jacobs School of Engineering, La Jolla, CA USA; 5 Department of Pathology, University of California San Diego School of Medicine, La Jolla, CA USA; 6 Department of Neurosciences, University of California San Diego School of Medicine, La Jolla, CA USA; 7 Halıcıoğlu Data Science Institute, University of California San Diego, La Jolla, CA USA; 8 Altman Clinical and Translational Research Institute, 9500 Gilman Drive, #0861, La Jolla, CA 92093 USA; 9 Department of Urology, University of California San Diego, La Jolla, CA USA

**Keywords:** Prostate, Cancer detection, *T*_2_ mapping, Restriction spectrum imaging, Diffusion-weighted imaging, Urological cancer, Cancer imaging, Tumour biomarkers, Urological cancer

## Abstract

The Restriction Spectrum Imaging restriction score (RSIrs) has been shown to improve the accuracy for diagnosis of clinically significant prostate cancer (csPCa) compared to standard DWI. Both diffusion and *T*_2_ properties of prostate tissue contribute to the signal measured in DWI, and studies have demonstrated that each may be valuable for distinguishing csPCa from benign tissue. The purpose of this retrospective study was to (1) determine whether prostate *T*_2_ varies across RSI compartments and in the presence of csPCa, and (2) evaluate whether csPCa detection with RSIrs is improved by acquiring multiple scans at different TEs to measure compartmental *T*_2_ (c*T*_2_). Data includes two cohorts scanned for csPCa with 3T multi-*b*-value diffusion-weighted sequences acquired at multiple TEs. c*T*_2_ values were computed from multi-TE RSI data and compared by compartment. CsPCa detection was compared between RSIrs and a logistic regression model (LRM) to predict the probability of csPCa using c*T*_2_ in combination with RSI measurements. Two-sample t-tests (*α* = 0.05) and the area under the receiver operating characteristic curve (AUC) were used for the statistical analyses. In both cohorts, *T*_2_ was different (*p* < 0.05) across the four RSI compartments (*C*_*1,*_* C*_*2,*_* C*_*3,*_* C*_*4*_). Voxel-level, cohort 1: *T*_2_ was different in csPCa for *C*_*1,*_* C*_*2,*_* C*_3_ (*p* < 0.001). Patient-level, cohort 1: *T*_2_ was different in csPCa patients in *C*_3_ (*p* = 0.02); cohort 2: *T*_2_ differed in csPCa patients in *C*_1_ (*p* = 0.01), *C*_3_ (*p* = 0.01) and *C*_*4*_ (*p* < 0.01). Voxel-level csPCa detection: c*T*_2_ did not improve discrimination over RSIrs alone (*p* = 0.9). Patient-level: RSIrs and the LRM performed better than diffusion alone (*p* < 0.001), but the difference in AUCs between RSIrs and the LRM was not significantly different (*p* = 0.54). In conclusion, significant differences in c*T*_2_ were observed between normal and cancerous prostatic tissue. With our data, however, consideration of c*T*_2_ in addition to diffusion did not significantly improve cancer detection performance.

## Introduction

Multiparametric magnetic resonance imaging (mpMRI) has become an important tool for the diagnosis of prostate cancer (PCa)^1^. MpMRI has proven to reduce unnecessary biopsies, mitigate overdiagnosis of clinically insignificant prostate cancer (indolent PCa), and enhance detection of clinically significant prostate cancer (csPCa, Grade Group ≥ 2)^2,3^. In standard reporting of prostate MRI (Prostate Imaging Reporting and Data System, PI-RADS v2.1^4^), diffusion-weighted imaging (DWI) and *T*_2_ -weighted imaging are the principal modalities used to detect csPCa. DWI measures the random movement of water molecules within tissues, aiding in the visualization of areas of restricted diffusion associated with hypercellular csPCa^5^. Meanwhile, *T*_2_ -weighted imaging provides detailed anatomical information and facilitates the visualization of abnormalities in prostate tissue. The combined analysis of these two sequences allows for a more in-depth evaluation of potential tumor lesions, thereby contributing to csPCa detection and characterization.

The challenge with the interpretation of conventional mpMRI lies in its inherent subjectivity and variability^6^. The interpretations of imaging data by different radiologists that rely on qualitative assessment alone leads to inconsistencies in the identification and characterization of csPCa lesions^7^. Interobserver variability significantly limits the accuracy and reliability of csPCa diagnosis^8^. To enhance diagnostic accuracy, there is a growing emphasis on the development and adoption of quantitative MRI approaches. Quantitative MRI aims to provide objective metrics of tissue properties associated with the probability of csPCa, offering the potential for more standardized and reproducible image assessment.

Restriction Spectrum Imaging (RSI) is a quantitative approach to DWI for csPCa detection and characterization. RSI scans are acquired at multiple *b*-values (diffusion weightings) to distinguish diffusion signal from tissue micro-compartments (intracellular water, extracellular hindered water, freely diffusing water, and flowing fluid)^9–12^. However, RSI models typically do not incorporate quantitative *T*_2_ measurements. On the other hand, studies using luminal water imaging (LWI) and hybrid multidimensional MRI have shown that tissue *T*_2_ can differ between prostate tissue compartments and provide diagnostic information that is complementary to diffusion^13,14^.

In this study, we acquired prostate RSI data at multiple echo times (TEs) to measure compartmental *T*_2_ in addition to diffusion. We aimed to determine whether compartmental *T*_2_ (i.e., *T*_2_ within each RSI micro-compartment) differs between cancerous and normal prostate tissue, and whether consideration of compartmental *T*_2_ in RSI yields improved detection of csPCa.

## Methods

### Study population

This study was approved by the University of California San Diego (UCSD) institutional review board (IRB). All research was performed in accordance with relevant guidelines and regulations. An FDA-cleared, commercial version of RSI is used routinely in our center as part of clinical routine. The first cohort included 46 patients scanned for suspected or known PCa between August and December of 2016 with multiple TEs as part of a quality improvement project to determine the best TE. The second cohort included patients who were scanned for suspected or known PCa between March 2021 and January of 2023 with a multi-TE RSI protocol per clinical routine (the need to obtain informed consent was waived by the UCSD IRB for secondary use of routine clinical data) or after informed written consent as part of a clinical trial on treatment response assessment (clinicaltrials.gov NCT04349501). Patients were excluded if they had received any treatment for PCa prior to the MRI acquisition or if a lesion with PI-RADS score ≥ 3 was detected on MRI but no biopsy information was available.

### Routine clinical evaluation

Patients in both cohorts underwent prostate MRI as part of routine clinical care for PCa, except for 38 patients in cohort 2 who were scanned as part of a prospective research study without clinical evaluation by a radiologist. MpMRI was performed according to PI-RADS guidelines, and interpretation was made per clinical routine using PI-RADS v2.1. Several patients, all from cohort 2, had PCa diagnosed on systematic biopsy without MRI, and then had an MRI with RSI before any treatment as part of a prospective study; PI-RADS scores are not available for these subjects. PI-RADS interpretation was done as part of clinical practice, but the original lesion segmentations were not available for the present study. Those segmentations are made in routine clinical practice using a proprietary software for biopsy that does not permit exporting the lesions. The radiologists in the present study segmented all biopsy-confirmed lesions, relying on the images themselves and the written description of those biopsy targets provided in the routine clinical PI-RADS reports. The presence of clinically significant prostate cancer (csPCa, grade group ≥ 2) was determined from biopsy results, typically systematic 12-core biopsy with additional targeted cores for suspicious lesions on MRI. Patients with PI-RADS lesions of 1 or 2 with no biopsy were considered negative for csPCa, in accordance with European Association of Urology (EAU) guidelines^15–17^.

### RSI data acquisition and processing

All patients were scanned with an expanded MRI protocol that included two multi-*b*-value RSI acquisitions performed with different TEs. MRI acquisition details are summarized in Table 1. All MR imaging was performed on a 3T clinical scanner (Discovery MR750; GE Healthcare, Waukesha, WI, USA), using a 32-channel phased-array coil over the pelvis. For each patient, two axial, multi-*b*-value DWI volumes were separately acquired using two different TEs: 80 ms and 100 ms for cohort 1, and 76 ms and 90 ms for cohort 2. All other parameters were the same between scans. In addition to the DWI volumes, a single *T*_2_-weighted volume was acquired for anatomical reference using the same scan coverage as the DWI volumes. MRI post-processing was performed using programs implemented in MATLAB R2022a (MathWorks, Natick, MA, USA^18^). DWI volumes were corrected to account for *B*_*0*_-inhomogeneities, gradient nonlinearities, eddy currents^19^, and image noise^10^. Samples at each *b*-value were averaged together. Image registration^20^ was applied to correct for patient motion between acquisitions.

**Table 1:** Acquisition details for DWI and *T*_2_-weighted image volumes. All MR imaging was performed on a 3T clinical scanner (Discovery MR750; GE Healthcare), using a 32-channel phased-array coil over the pelvis. The two DWI volumes for both cohorts were acquired using different TEs to allow for examination of *T*_2_ relaxation in prostatic tissue compartments. The single *T*_2_-weighted volume was acquired for anatomical reference. *Diffusion-weighted echo-planar imaging. †Fast spin echo. ‡An extra* b* = 0 ${{\text{s}}/{\text{mm}}}^{2}$ volume was acquired with reverse phase encoding to enable correction of *B*_*0*_-inhomogeneity distortions.

Cohort 1	DWI 1	DWI 2	*T*_2_-weighted
Pulse sequence	EPI*	EPI*	FSE†
TR (ms)	5000	5000	6225
TE (ms)	80	100	100
FOV (mm)	220 × 220	220 × 220	220 × 220
Matrix [resampled dimensions]	96 × 96 [128 × 128]	96 × 96 [128 × 128]	320 × 320 [512 × 512]
Slices	34	34	34
Slice Thickness (mm)	3	3	3
*b*-values (s/mm^2^) [number of samples]	0[7‡], 200 [6], 1000 [6], 2000 [6], 3000 [6]	0[7‡], 200 [6], 1000 [6], 2000 [6], 3000 [6]	N/A

Cohort 2	DWI 1	DWI 2	*T*_2_-weighted
Pulse sequence	EPI*	EPI*	FSE†
TR (ms)	4500	4500	6230
TE (ms)	76	90	98
FOV (mm)	20 × 100	200 × 100	200 × 200
Matrix [resampled dimensions]	80 × 48 [128 × 128]	80 × 48 [128 × 128]	320 × 320 [512 × 512]
Slices	32	32	32
Slice Thickness (mm)	3	3	3
*b*-values (s/mm^2^) [number of samples]	0 [2‡], 50[6], 800[6], 1500[12], 3000 [18]	0 [2‡], 50[6], 800[6], 1500[12], 3000 [18]	N/A

For patients in cohort 1, regions of interest (ROIs) were manually defined on *T*_2_-weighted images over the whole prostate, peripheral zone, and transition zone (the central zone was included with the transition zone). The contouring of the prostate zones and tumor lesions was performed using MIM software (MIM software version 7.2.6, Inc; Cleveland, OH, USA^21^), by a radiation oncologist with 3 years of experience and two board-certified sub-specialist radiologists with 4 and 6 years of experience, using all available clinical imaging and pathologic information^11^. Radiologist-certified contours of the prostate zones and lesions were not obtainable for cohort 2. Instead, automated prostate contours, which are generally highly accurate^22^, were obtained using an FDA-cleared commercial product (OnQ Prostate version 1.4, CorTechs.ai, San Diego, CA, USA^23^).

### RSI modeling

Prior studies established and validated a four-compartment RSI model of the diffusion signal^10,11,24^:

$$S\left( b \right) = \sum\limits_{{i = 1}}^{4} {C_{i} e^{{ - bD_{i} }} }$$

*S(b)* denotes the measured DWI signal intensity at a particular *b*-value, which is modeled as a linear combination of exponential decays representing four diffusion compartments. *C*_*i*_ describes the compartmental signal contributions to be determined via model-fitting. The diffusion coefficients, *D*_*i*_, are fixed for each of the four tissue compartments to empirically determined values^10^ that broadly represent restricted diffusion, hindered diffusion, free diffusion, and vascular flow: 1.1e−4, 1.8e−3, 3.6e−3, and 0.1220 mm^2^/s, respectively. Signal-contribution (*C*_*i*_) maps were computed for both DWI volumes per patient by fitting this model to the signal-vs.-*b*-value curve from each voxel. A previously validated biomarker for PCa called the RSI restriction score (RSIrs) was computed by dividing the signal intensity of the restricted diffusion compartment, *C*_1_, at each voxel by the median signal intensity within the whole prostate on the *b* = 0 mm^2^/s DWI images (an index of apparent *T*_2_-weighting in the prostate)^11,24–27^.

### Compartmental *T*_2_ mapping and analysis by csPCa status

*T*_2_ maps were computed for each compartment of the RSI model by fitting the monoexponential *T*_2_ decay formula to the signal values from the two *C*_*i*_ maps with different TEs. The median *T*_2_ within the whole prostate was then computed. Two-sample *t*-tests (*α* = 0.05) were used to determine whether there were significant differences in median *T*_2_ between compartments.

For cohort 1, two-sample *t*-tests (*α* = 0.05) were used to compare median compartmental *T*_2_ values between benign or clinically insignificant PCa tissue and csPCa lesions. For both cohorts, we used two-sample *t*-tests (*α* = 0.05) to compare each patient’s median *T*_2_ by compartment in the whole prostate, and whether compartmental *T*_2_ was significantly different between patients with and without csPCa. Any compartments with a significant difference in *T*_2_ between normal and cancerous tissue were noted for inclusion in subsequent multivariable modeling.

### Logistic regression model fitting and evaluation of cancer-detection performance

A logistic regression model (LRM) was developed to estimate the probability that a given voxel of tissue contains csPCa given measurements of diffusion and compartmental *T*_2_. RSIrs^11^ was included as the diffusion parameter of the model. Compartmental *T*_2_ was included in the LRM for each compartment that showed a significant difference in *T*_2_ between normal and cancerous tissue. Cohort 1 had radiologist-certified lesion contours available and was therefore used to train the LRM. In patients with csPCa, voxels inside the lesion contours were labeled as csPCa-positive, while prostate voxels outside the lesion contours were labeled as csPCa-negative. In these patients, voxels labeled as csPCa-positive were included to train the LRM and all non-csPCa voxels were excluded. In patients without csPCa, diffusion and compartmental *T*_2_ measurements from all voxels within the entire prostate were used to train the LRM and labeled as csPCa-negative.

Ten-fold cross-validation was performed to evaluate voxel-level csPCa-detection performance of the model within cohort 1. We assessed csPCa-detection performance using the area under the receiver operating characteristic curve (AUC) and calculated 95% confidence intervals (CI) from 10,000 bootstrap samples.

Both cohorts were used to test the patient-level csPCa-detection performance of the model. For patient-level analysis of the LRM, the highest probability value observed within the whole prostate was used as the predictor variable. Similarly, the maximum RSIrs value within the whole prostate was used as the patient-level predictor for RSIrs. We also computed maximum *C*_1_ to obtain the patient-level performance of diffusion only, as RSIrs incorporates global prostate *T*_2_ signal in addition to diffusion signal. AUC values were computed for maximum *C*_1_, maximum RSIrs, and the LRM, and compared using two-sample *t*-tests (*α* = 0.05). The 95% confidence intervals were estimated through random sampling with replacement from 10,000 bootstrap patient samples.

## Results

### Study population

Cohort 1 comprised 46 patients (age: 70 ± 10 years; PSA: 10.6 ± 16.9 ng/mL). Cohort 2 comprised 195 patients (age: 69 ± 8 years; PSA: 8.2 ± 8.5 ng/mL). In cohort 1, 22 of 46 patients (47.8%) had csPCa, while the remaining 24 had either low-grade (grade group 1) disease or no cancer. In cohort 2, 96 of 195 (49.2%) patients had csPCa. 38 participants from cohort 2 had no PI-RADS scores available because csPCa was diagnosed on systematic biopsy without MRI and then had an MRI with RSI acquisition as part of a separate, prospective study. Table 2 summarizes the patient characteristics of both cohorts.

**Table 2:** Summary of radiologic and pathologic characteristics of the two cohorts of patients included in this study. *4 patients had PI-RADS 1 scores, and 1 patient had PI-RADS 2 scores. **27 patients had PI-RADS 1 scores, and 4 patients had PI-RADS 2 scores. †Part of a prospective research study without clinical evaluation by a radiologist.

Cohort 1 (n = 46)		
Available pathology	Systematic biopsy only	6
	Targeted Biopsy Only	0
	Systematic and targeted biopsy	20
	Prostatectomy	15
	No biopsy or prostatectomy*	5
PI-RADS	1	9
	2	2
	3	10
	4	10
	5	15
	Not available	0
Gleason grade group	Benign	17
	1	2
	2	9
	3	8
	4	1
	5	4

Cohort 2 (n = 195)		
Available pathology	Systematic biopsy only	44
	Targeted Biopsy Only	5
	Systematic and targeted biopsy	60
	Prostatectomy	28
	No biopsy or prostatectomy**	31
PI-RADS	1	66
	2	10
	3	18
	4	36
	5	27
	Not available†	38
Gleason grade group	Benign	38
	1	30
	2	40
	3	32
	4	7
	5	17

### Compartmental *T*_2_ mapping

**Fig. 1:**
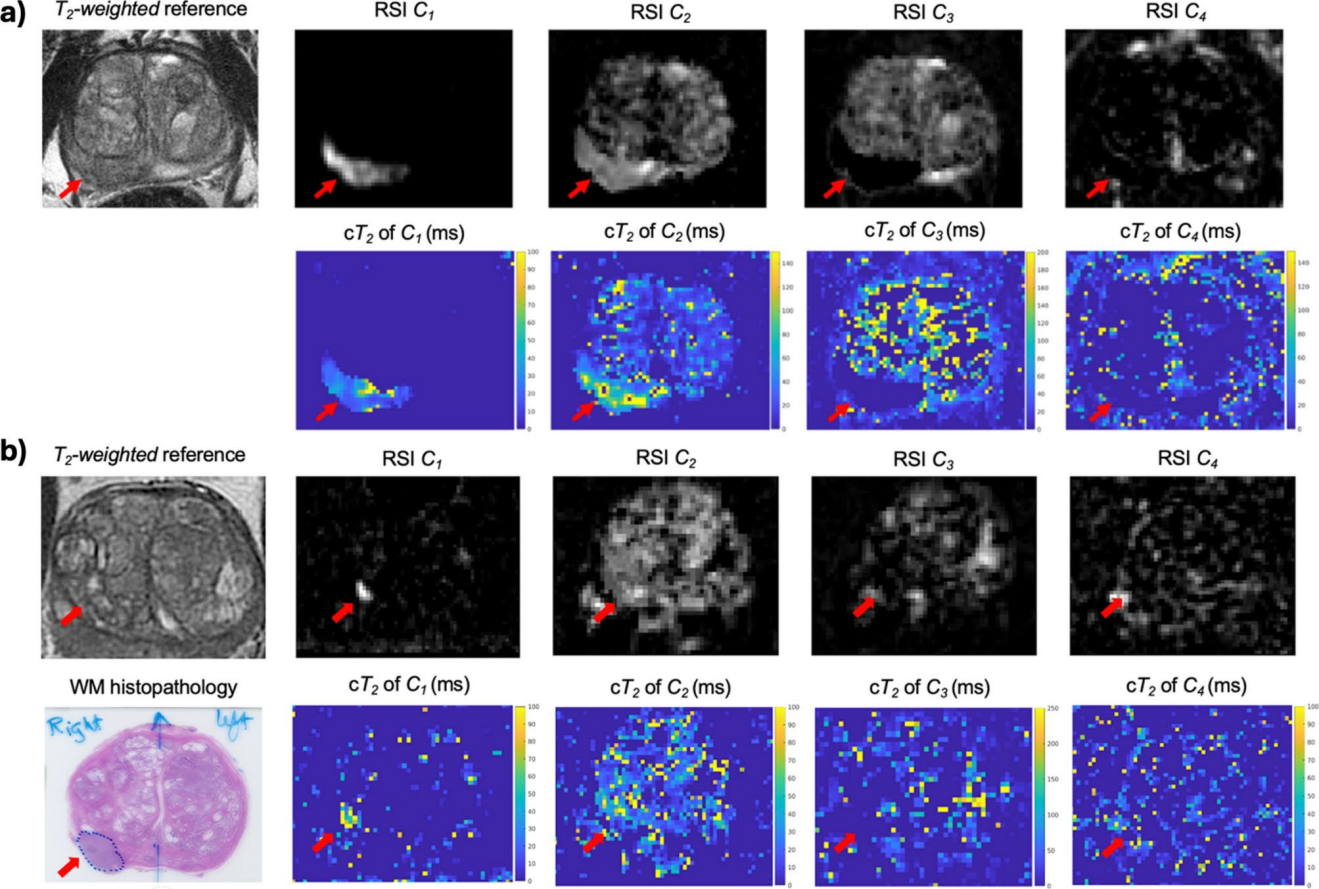
RSI signal contribution (*C*_*i*_) and compartmental *T*_2_ (c*T*_2_) maps for two patients with csPCa. (**A**) Patient from cohort 1. (**B**) Patient from cohort 2. Compartmental *T*_2_ maps were computed for each RSI model compartment by measuring the *T*_2_ -weighted signal decay of the signal-contribution map for different TEs. The signal-contribution maps shown here were computed from the DWI acquisition with shorter TE. Whole-mount (WM) histopathology results were available for the patient from cohort 2 and illustrate the lesion contour in the prostate. For cohort 1, the contouring of the prostate zones and tumor lesions was performed using MIM software (MIM software version 7.2.6, Inc; Cleveland, OH, USA^21^). For cohort 2, automated prostate contours were obtained using an FDA-cleared commercial product (OnQ Prostate version 1.4, CorTechs.ai, San Diego, CA, USA^23^).

Figure 1 shows compartmental *T*_2_ maps for two patients with csPCa, one from each cohort.

**Fig. 2:**
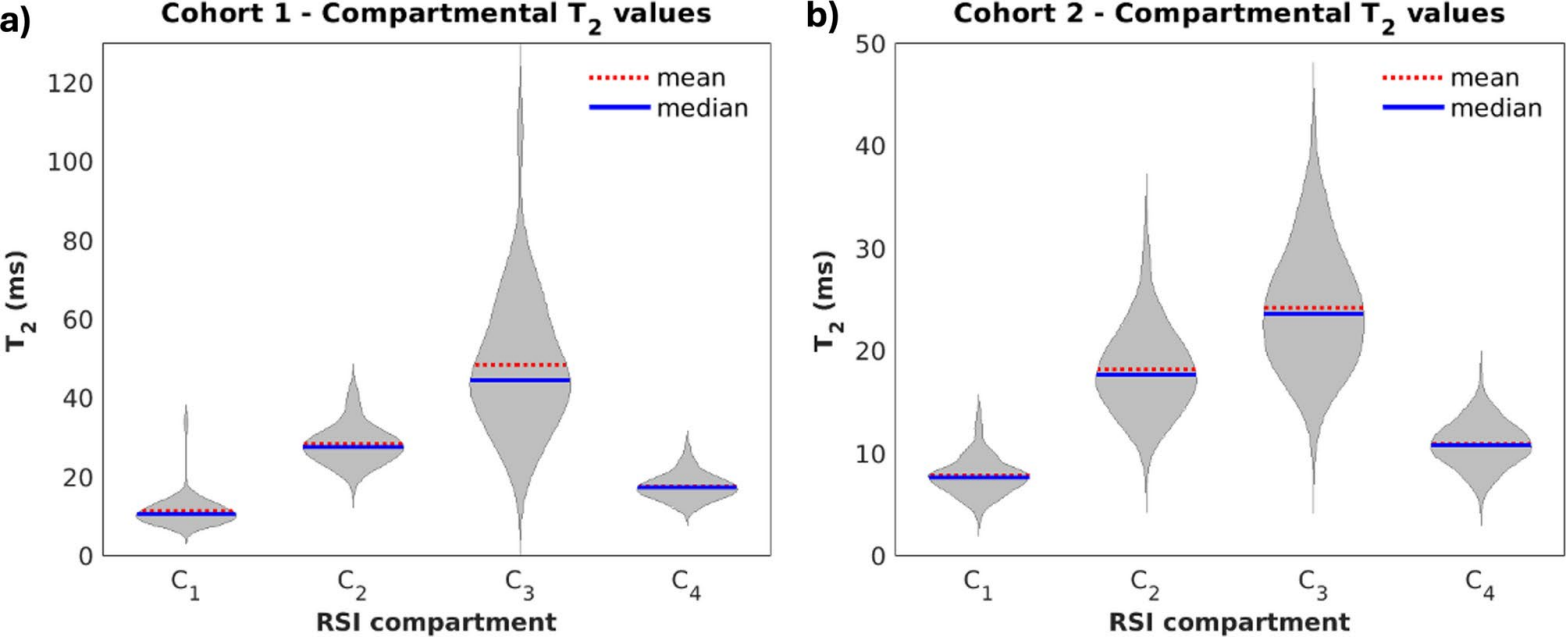
Violin plots showing the distribution of median *T*_2_ values in the whole prostate for each of the four RSI model compartments. Within each cohort, compartmental *T*_2_ was significantly different between any two compartments (*p* < 0.05). (**A**) cohort 1 (n = 46). (**B**) cohort 2 (n = 195). Plots were created using MATLAB R2022a (MathWorks, Natick, MA, USA^18^). For cohort 1, the contouring of the prostate zones and tumor lesions was performed using MIM software (MIM software version 7.2.6, Inc; Cleveland, OH, USA^21^). For cohort 2, automated prostate contours were obtained using an FDA-cleared commercial product (OnQ Prostate version 1.4, CorTechs.ai, San Diego, CA, USA^23^).

Figure 2 shows violin plots of median *T*_2_ within the whole prostate for each RSI model compartment. In both cohorts, the highest median *T*_2_ values were observed in *C*_3_, followed by *C*_2_, *C*_*4*_, and finally *C*_1_. Compartmental *T*_2_ was significantly different between any two compartments (*p* < 0.05).

### Voxel-level analysis of compartmental *T*_2_ values by csPCa status

**Fig. 3:**
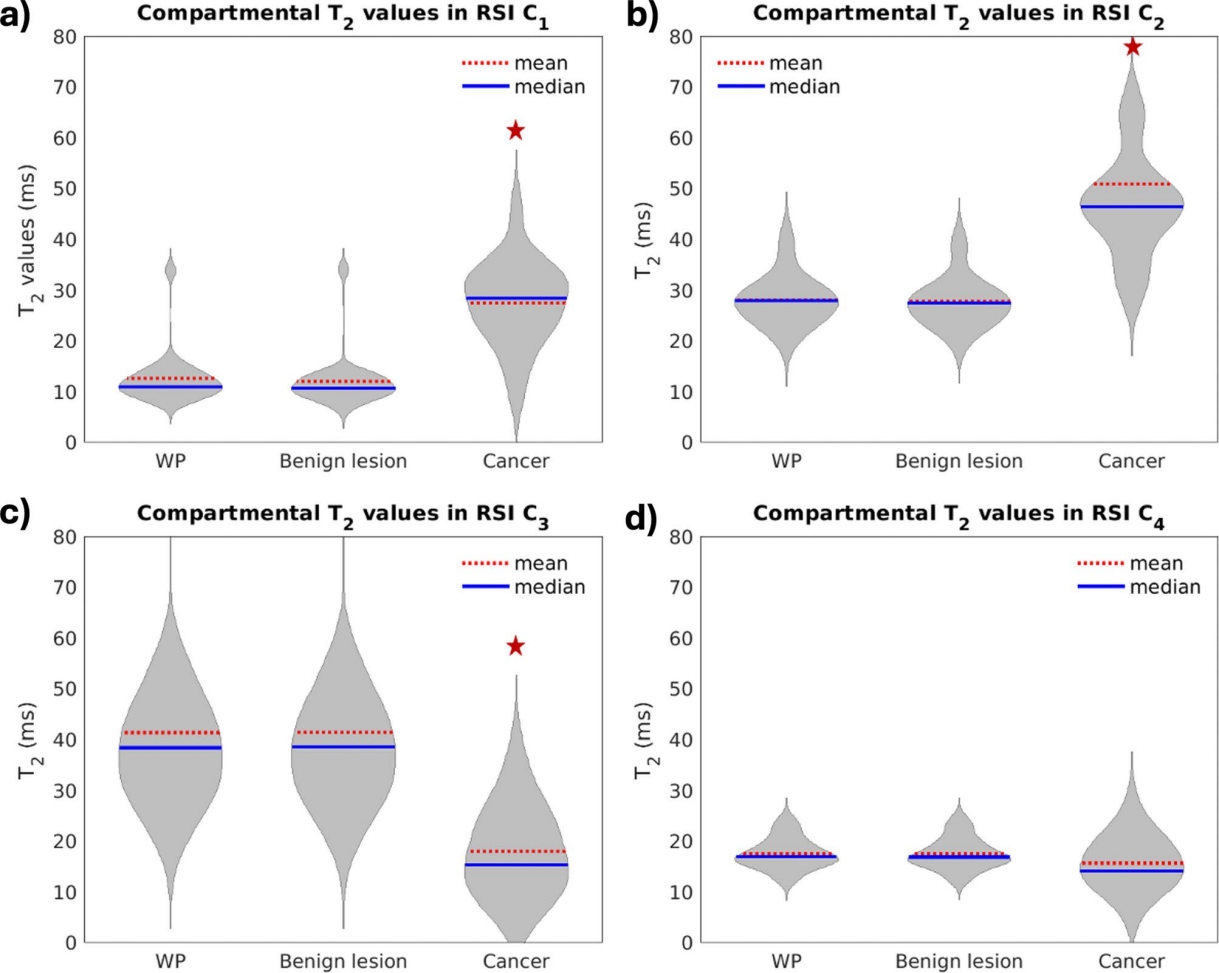
Violin plots showing the distribution of median compartmental *T*_2_ values from cohort 1 in both csPCa lesions and the surrounding prostate tissue. Each panel corresponds to one of the RSI diffusion compartments. csPCa lesions showed significantly higher compartmental T2 values in *C*_1_ (*p*-value <  < 0.001) and *C*_2_ (*p*-value <  < 0.001) than tissues outside of csPCa lesions. Compartmental *T*_2_ values of *C*_3_ were also significantly lower in csPCa lesions than outside csPCa lesions (*p*-value <  < 0.001). A red star indicates a significant difference (*p-*value < 0.05) in compartmental *T*_2_ between csPCa lesions and the prostate tissue outside the lesions. WP: whole prostate. Plots were created using MATLAB R2022a (MathWorks, Natick, MA, USA^18^). For cohort 1, the contouring of the prostate zones and tumor lesions was performed using MIM software (MIM software version 7.2.6, Inc; Cleveland, OH, USA^21^).

For each compartment of the RSI model, the comparison of *T*_2_ values between csPCa and prostate tissue outside of csPCa lesions is illustrated in Fig. 3. This figure corresponds to cohort 1, which has radiologist-certified csPCa lesion contours. csPCa lesions showed significantly higher compartmental *T*_2_ values in compartment 1 (*p* << 0.001) and compartment 2 (*p* < < 0.001) than normal tissue. In addition, the compartmental *T*_2_ values of compartment *C*_3_ were significantly lower in csPCa lesions (*p* << 0.001). The compartmental *T*_2_ values for compartment *C*_4_ were not significantly different between csPCa and normal tissues (*p* = 0.17).

### Patient-level analysis of compartmental *T*_2_ values by csPCa status

**Fig. 4:**
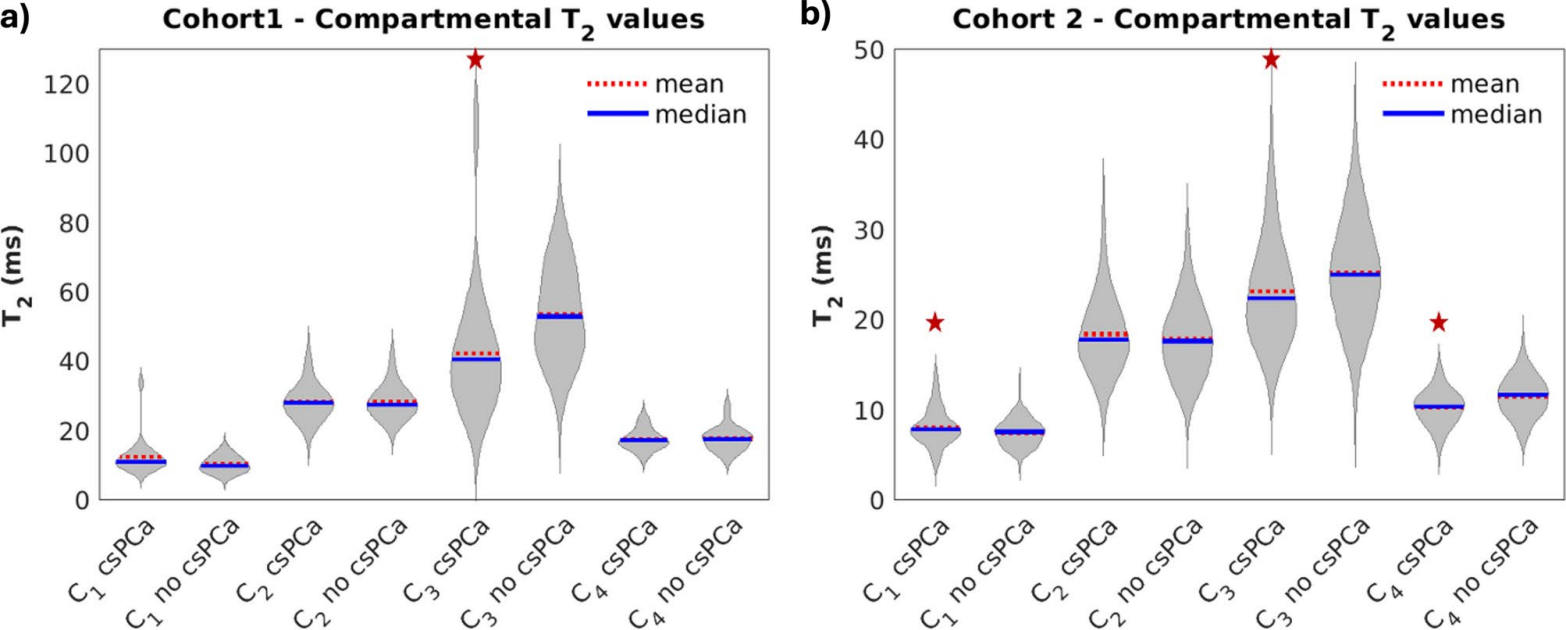
Violin plots comparing compartmental *T*_2_ within the whole prostate between patients with csPCa and those without. A red star indicates a significant difference (*p-*value < 0.05) in whole-prostate compartmental *T*_2_ between csPCa and non-csPCa patients. (**A**) cohort 1 (n = 46). (**B**) cohort 2 (n = 195). Plots were created using MATLAB R2022a (MathWorks, Natick, MA, USA^18^). For cohort 1, the contouring of the prostate zones and tumor lesions was performed using MIM software (MIM software version 7.2.6, Inc; Cleveland, OH, USA^21^). For cohort 2, automated prostate contours were obtained using an FDA-cleared commercial product (OnQ Prostate version 1.4, CorTechs.ai, San Diego, CA, USA^23^).

The comparison of compartmental *T*_2_ values between patients with and without csPCa is shown in Fig. 4. In both cohorts, patients with csPCa had higher compartmental *T*_2_ values in compartment *C*_1_ than patients with no csPCa. In cohort 2, median *C*_1_ compartmental *T*_2_ was significantly higher (*p* = 0.07 for cohort 1; *p* = 0.01 for cohort 2). Median *C*_3_ compartmental *T*_2_ was significantly different between csPCa and patients without csPCa in both cohorts (*p* = 0.02 for cohort 1; *p* = 0.01 for cohort 2). Median *C*_*4*_ compartmental *T*_2_ was also significantly different in cohort 2 (*p* < < 0.01). Compartmental *T*_2_ values for the other compartments were not significantly different between patients with and without csPCa.

### Logistic regression model fitting and evaluation of csPCa-detection performance

**Fig. 5:**
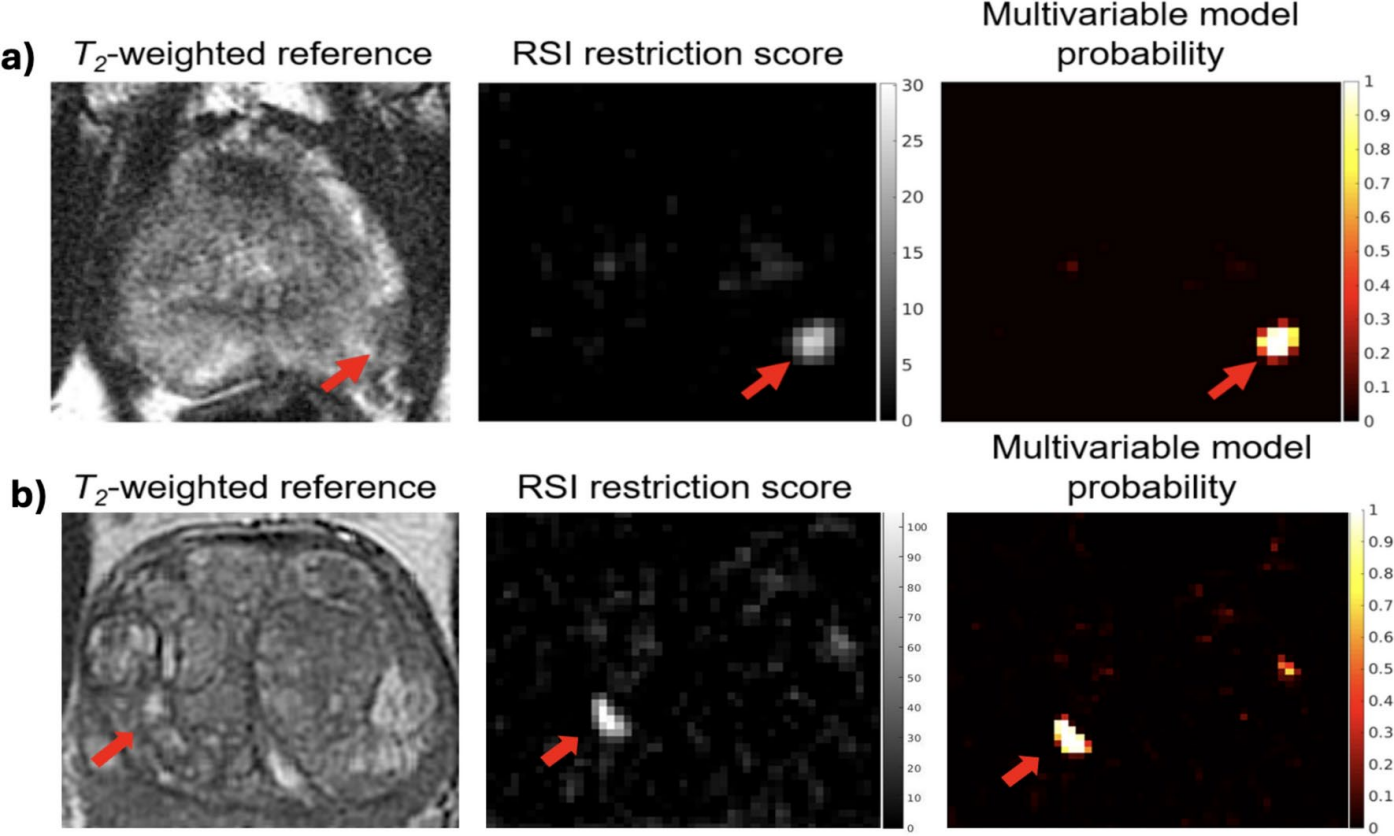
RSI restriction score and multivariable model probability maps of the prostate for patients with csPCa. Top panel: Patient from cohort 1 with a lesion in the left peripheral zone. Bottom panel: Patient from cohort 2 with a lesion in the transition zone. The multivariable model uses compartmental *T*_2_ measurements from each voxel in addition to the RSI restriction score to determine the probability that it contains csPCa. For the patient from cohort 1, the contouring of the prostate zones and tumor lesions was performed using MIM software (MIM software version 7.2.6, Inc; Cleveland, OH, USA^21^). For patient from cohort 2, automated prostate contours were obtained using an FDA-cleared commercial product (OnQ Prostate version 1.4, CorTechs.ai, San Diego, CA, USA^23^).

*T*_2_ measurements from RSI compartments 1, 2, and 3 (*C*_1_, *C*_2_, *C*_3_) were included as the *T*_2_ parameters of the LRM. These three compartments showed significantly different median *T*_2_ signal between csPCa lesions vs csPCa-negative voxels. The LRM predictors were RSIrs, *C*_1_-*T*_2_, *C*_2_*-T*_2_, and *C*_3_-*T*_2_. The model coefficients with 95% confidence interval for the y-intercept and RSIrs were 6.367 (6.323, 6.416) and -51.621 (-52.354, -50.888), respectively. The weights for C_1_*-T*_2_, *C*_2_*-T*_2_, and *C*_3_* T*_2_ were < 0.005. Example probability maps computed from the model are shown in Fig. 5 for two patients with csPCa, alongside maps of RSIrs.

### Voxel-level cancer detection

For voxel-level cancer detection, the tenfold cross-validation mean AUC of the LRM was 0.98 [95% CI: 0.957–0.985], versus 0.98 [95% CI: 0.958–0.986] for maximum RSIrs, indicating that incorporating compartmental *T*_2_ did not improve discrimination over RSIrs alone (*p* = 0.9).

### Patient-level cancer detection

For cohort 1, the AUC of the LRM was 0.804 [0.648–0.930], versus 0.805 [0.648–0.931] for RSIrs. The mean AUC for maximum *C*_1_ was 0.695 [0.530–0.851]. The difference in AUCs between RSIrs and the multivariable model was not significantly different (*p* = 0.26). Both RSIrs and the LRM performed significantly better than diffusion (maximum *C*_1_) alone (both *p* << 0.001).

**Fig. 6:**
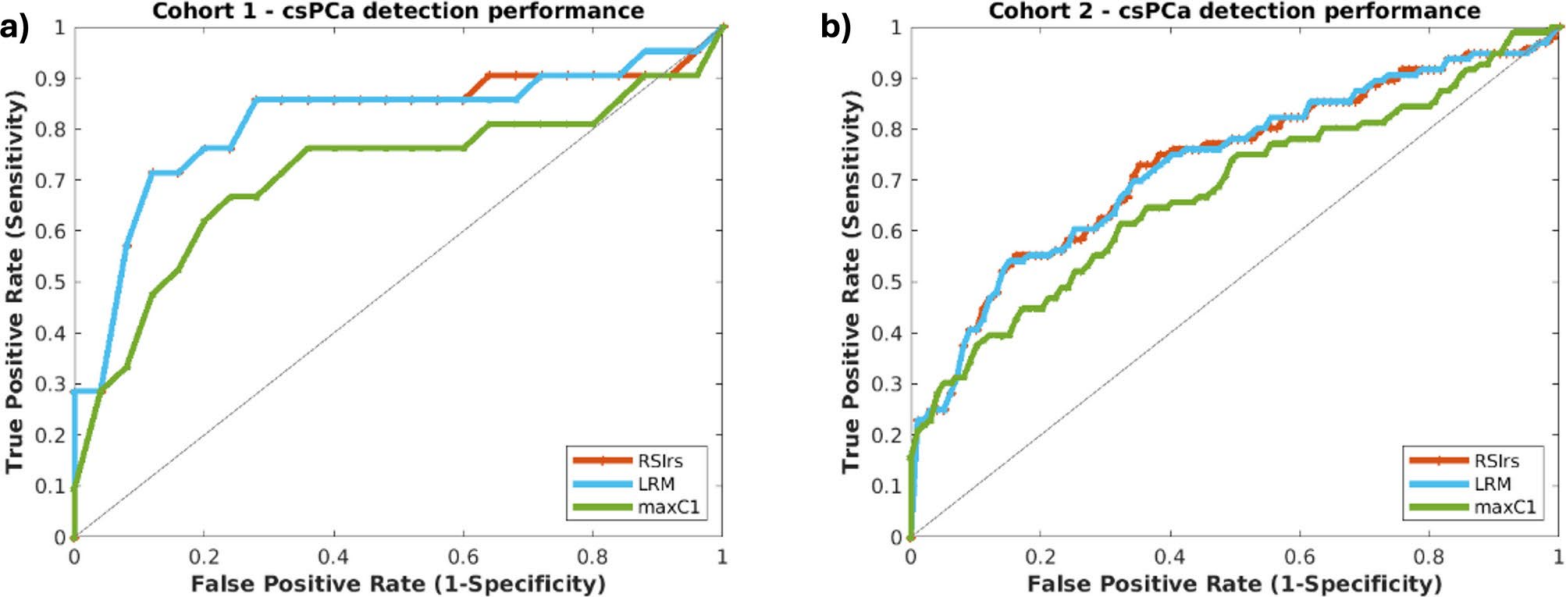
Patient-level diagnostic performance of maximum RSIrs, the LRM and maximum *C*_1_ (max* C*_1_) for cohorts 1 and 2. (**A**) For cohort 1, mean AUC values were 0.695 for maximum *C*_1_, 0.805 for RSIrs and 0.804 for the multivariable model. (**B**) For cohort 2, mean AUC values were 0.654 for maximum *C*_1_, 0.725 for RSIrs and 0.724 for the multivariable model. Plots were created using MATLAB R2022a (MathWorks, Natick, MA, USA^18^). For cohort 1, the contouring of the prostate zones and tumor lesions was performed using MIM software (MIM software version 7.2.6, Inc; Cleveland, OH, USA^21^). For cohort 2, automated prostate contours were obtained using an FDA-cleared commercial product (OnQ Prostate version 1.4, CorTechs.ai, San Diego, CA, USA^23^).

For cohort 2, the mean LRM AUC for 10,000 bootstrapped samples was 0.724 [0.650–0.793]. The mean AUC of RSIrs for 10,000 bootstrapped samples was 0.725 [0.652–0.794]. For maximum *C*_1_ the mean AUC was 0.654 [0.573, 0.730]. The difference in AUCs between RSIrs and the multivariable model was not significantly different (*p* = 0.54). Both RSIrs and the LRM performed significantly better than diffusion (maximum *C*_1_) alone (both *p* << 0.001). ROC curves for both cohorts are shown in Fig. 6.

## Discussion

We found that compartmental *T*_2_ values were significantly different across RSI diffusion compartments. Moreover, compartmental *T*_2_ values differed between csPCa lesions and benign tissue or low-grade PCa in RSI compartments C_*1,*_ C_*2,*_ and C_3_. At the patient level, there were also differences in whole-prostate *T*_2_ between patients with no csPCa and those with biopsy-proven csPCa. Quantitative differences in compartmental *T*_2_ may provide insight into the microstructural changes associated with PCa. For example, extracellular matrix remodeling may contribute to the increased compartmental *T*_2_ observed in *C*_2_^12^. Lower compartmental *T*_2_ values in C_3_ may reflect hyperplasia-induced reductions in luminal space^13^. While the elevated compartmental *T*_2_ observed in *C*_1_ of csPCa patients may seem opposed to the known overall hypointense appearance of csPCa on *T*_2_-weighted MRI, it is consistent with an increase in nuclear volume fraction that is typical of cancer cells^28^.

We have previously shown that RSIrs is a useful quantitative DWI biomarker for csPCa at the voxel- and patient-level^11,24,26^. Here, we evaluated whether incorporating compartmental *T*_2_ values would improve csPCa discrimination over RSIrs alone. We demonstrated that *T*_2_ effects have csPCa discriminatory value, as they showed higher AUC values compared to diffusion alone in both cohorts. However, consideration of compartmental *T*_2_ did not significantly improve csPCa-detection performance over maximum RSIrs at the voxel- or patient-level. This finding may suggest an overlap of microstructural information that is captured by diffusion and *T*_2_-weighted imaging. A number of studies have demonstrated an interdependence between apparent diffusion coefficient (ADC) measurements and *T*_2_ values^29,30^. Diffusion measurements from RSI and *T*_2_ measurements from techniques like Luminal Water Imaging (LWI) are both also strongly correlated with microstructural tissue features, including cellularity and luminal water space, that are indicative of cancer^31^. It may be that the RSI and *T*_2_ measurements in this study both reflect similar aspects of tissue microstructure, and their combination therefore does not yield a substantial increase of diagnostically useful information^32^.

As with any conventional DWI acquisition, signal from the individual RSI compartments is also partially *T*_2_-weighted. RSIrs further incorporates a measure of global *T*_2_-weighted signal in the prostate, namely the median signal within the prostate on the *b* = 0 s/mm^2^ volume. These *T*_2_-weighting factors intrinsic to RSIrs are correlated with the values obtained from quantitative *T*_2_-mapping, so the addition of *T*_2_-mapping may not have provided sufficiently complementary information to improve csPCa discrimination performance.

Other advanced imaging techniques have also been used to measure *T*_2_ in the prostate. LWI utilizes the unique *T*_2_ relaxation rates associated with various components of prostate tissue to quantify the fractional volume of glandular lumen, denoted as luminal water fraction (LWF)^33^. This method takes advantage of the observed alterations in the composition of prostatic tissue in the presence of cancer and the Gleason grade of the cancer for PCa diagnosis^31^. Hybrid multidimensional MRI (HM-MRI) exploits the interdependence of *T*_2_ and ADC values to measure prostate volume fractions of the lumen, epithelium, and stroma^34^. Studies using LWI and HM-MRI demonstrate lower *T*_2_ values in cancer lesions compared to normal prostate tissue with increasing Gleason Grade. The decrease in *T*_2_ results from a decrease in luminal volume due to cellular hyperplasia^31,35^. This trend agrees with the decrease observed in this study of *T*_2_ in compartment *C*_3_ of patients with csPCa. This RSI compartment reflects signal from freely diffusing water in the prostate, which we expect to find predominantly in luminal tissue and to be impacted by a reduction in luminal space. Prior work with HM-MRI showed that the decrease in luminal space is largely the result of epithelial tissue proliferation, indicated by an increase in the measured epithelial volume fraction^34^. Since RSI does not explicitly assign signal contributions to a particular tissue type, this aspect of HM-MRI is harder to align with the present study. However, we can be sure that the changes observed in the *T*_2_ of compartment *C*_1_ reflect, at least in part, an increase in the overall tissue cellularity^36^. Signal contributions in this compartment are also dependent upon the nuclear volume fraction of cells in the tissue^28^, and the increase in the *T*_2_ observed in *C*_1_ of csPCa patients suggests an increase in nuclear volume fraction.

While quantitative *T*_2_ did not yield improved patient-level csPCa discrimination compared to RSI alone, the ROI-based analysis of this study suggests that it may be helpful for lesion-level detection and characterization of tumors at a microscopic level. Whole-mount histopathology (WMHP) data are currently being collected as part of an ongoing study to map changes in compartmental RSI signal to the histological restructuring of prostate tissue due to csPCa. This mapping aims to correlate RSI and *T*_2_ signal with specific microscopic alterations observed in tumors. This approach could also aid in identifying variations in *T*_2_ across different tumor grades, providing a non-invasive means to predict grade group and assess the aggressiveness of cancer^13^, a crucial prognostic indicator. Another ongoing, prospective trial (ART-Pro; NCT06579417) is evaluating the impact of RSIrs and compartmental *T*_2_ on csPCa diagnosis in a heterogeneous multi-center and multi-reader dataset^37^.

Future work that pairs RSI with a more advanced, multicompartmental approach to *T*_2_ measurement, such as LWI, would allow for deeper insight into the relationship between compartmental diffusion and *T*_2_ than was achievable here. Our acquisition protocol involved only a sparse sampling of two TEs compared to 5 *b*-values, while LWI would provide many more TE measurements to enable a more granular assessment of the *T*_2_ microenvironment. This would help determine whether diffusion and *T*_2_ measurements truly provide complementary information about the prostate and serve to enhance csPCa detection accuracy when used together.

## Limitations

The use of separate acquisitions and only two TEs per patient may have limited the accuracy of voxel-wise compartmental *T*_2_ measurements. However, the inclusion of additional TEs was restricted to avoid excessive protocol length on active clinical scanners.

While other quantitative MRI approaches use lower TEs (< 30 ms) for *T*_2_ mapping^14,31^, the high *b*-values required by RSI to optimally estimate *C*_1_ are generally incompatible with very low TEs on clinical scanners. Separate acquisitions would therefore be required to evaluate the combination of optimal RSIrs and *T*_2_ estimated with very low TE. Another limitation of this study is that our voxel-level data included only high-confidence csPCa and control categories, leaving little room for improvement over RSIrs for the voxel-level analysis^11^.

## Conclusion

*T*_2_ mapping affords insights into characteristics of benign and cancerous prostate tissue, but we did not find compelling evidence that acquisitions with multiple TE is necessary for patient-level csPCa detection with RSI.

## Data Availability

Data are available for bona fide researchers who request it from the authors. Please contact Dr. Tyler Seibert (tseibert@health.ucsd.edu) if you would like to request the data from this study.

## References

[1] Leung, D.K.-W., Chiu, P.K.-F., Ng, C.-F. & Teoh, J.Y.-C. Role of pre-biopsy multiparametric MRI in prostate cancer diagnosis: Evidence from the literature. *Turk. J. Urol.* **47**(Suppl 1), S65–S70. 10.5152/tud.2020.20360 (2021). 33016871 10.5152/tud.2020.20360 PMC8057361

[2] de Rooij, M., Hamoen, E. H. J., Fütterer, J. J., Barentsz, J. O. & Rovers, M. M. Accuracy of multiparametric MRI for prostate cancer detection: A meta-analysis. *AJR Am. J. Roentgenol.* **202**(2), 343–351. 10.2214/AJR.13.11046 (2014). 24450675 10.2214/AJR.13.11046

[3] Schoots, I. G. et al. Magnetic resonance imaging-targeted biopsy may enhance the diagnostic accuracy of significant prostate cancer detection compared to standard transrectal ultrasound-guided biopsy: A systematic review and meta-analysis. *Eur. Urol.* **68**(3), 438–450. 10.1016/j.eururo.2014.11.037 (2015). 25480312 10.1016/j.eururo.2014.11.037

[4] Scott, R., Misser, S. K., Cioni, D. & Neri, E. PI-RADS v2.1: What has changed and how to report. *SA J. Radiol.* **25**(1), 2062. 10.4102/sajr.v25i1.2062 (2021). 34230862 10.4102/sajr.v25i1.2062 PMC8252188

[5] Demirel, H. C. & Davis, J. W. Multiparametric magnetic resonance imaging: Overview of the technique, clinical applications in prostate biopsy and future directions. *Turk. J. Urol.* **44**(2), 93–102. 10.5152/tud.2018.56056 (2018). 29511576 10.5152/tud.2018.56056 PMC5832385

[6] Pickersgill, N. A. et al. Accuracy and variability of prostate multiparametric magnetic resonance imaging interpretation using the prostate imaging reporting and data system: A blinded comparison of radiologists. *Eur. Urol. Focus* **6**(2), 267–272. 10.1016/j.euf.2018.10.008 (2020). 30327280 10.1016/j.euf.2018.10.008

[7] Midiri, F., Vernuccio, F., Purpura, P., Alongi, P. & Bartolotta, T. V. Multiparametric MRI and radiomics in prostate cancer: A review of the current literature. *Diagn. Basel Switz.* **11**(10), 1829. 10.3390/diagnostics11101829 (2021). 10.3390/diagnostics11101829 PMC8534893 34679527

[8] Stabile, A. et al. Factors influencing variability in the performance of multiparametric magnetic resonance imaging in detecting clinically significant prostate cancer: A systematic literature review. *Eur. Urol. Oncol.* **3**(2), 145. 10.1016/j.euo.2020.02.005 (2020). 32192942 10.1016/j.euo.2020.02.005 PMC8942295

[9] Brunsing, R. L. et al. Restriction spectrum imaging: An evolving imaging biomarker in prostate MRI: Prostate MRI with restriction spectrum imaging: A review. *J. Magn. Reson. Imaging* **45**(2), 323–336. 10.1002/jmri.25419 (2017). 27527500 10.1002/jmri.25419 PMC5222783

[10] Conlin, C. C. et al. Improved characterization of diffusion in normal and cancerous prostate tissue through optimization of the restriction spectrum imaging signal model. *Radiol. Imaging*10.1101/2020.03.27.20042069 (2020). 10.1002/jmri.27393 PMC8178435 33131186

[11] Feng, C. H. et al. Voxel-level classification of prostate cancer on magnetic resonance imaging: Improving accuracy using four-compartment restriction spectrum imaging. *J. Magn. Reson. Imaging* **54**(3), 975–984. 10.1002/jmri.27623 (2021). 33786915 10.1002/jmri.27623 PMC8363567

[12] White, N. S. et al. Diffusion-weighted imaging in cancer: Physical foundations and applications of restriction spectrum imaging. *Cancer Res.* **74**(17), 4638–4652. 10.1158/0008-5472.CAN-13-3534 (2014). 25183788 10.1158/0008-5472.CAN-13-3534 PMC4155409

[13] Hectors, S. J., Said, D., Gnerre, J., Tewari, A. & Taouli, B. Luminal water imaging: Comparison With diffusion-weighted imaging (DWI) and PI-RADS for characterization of prostate cancer aggressiveness. *J. Magn. Reson. Imaging* **52**(1), 271–279. 10.1002/jmri.27050 (2020). 31961049 10.1002/jmri.27050

[14] Chatterjee, A. et al. Diagnosis of prostate cancer with noninvasive estimation of prostate tissue composition by using hybrid multidimensional MR imaging: A feasibility study. *Radiology* **287**(3), 864–873. 10.1148/radiol.2018171130 (2018). 29393821 10.1148/radiol.2018171130 PMC5978456

[15] EAU Guidelines on Prostate Cancer—Uroweb. Uroweb - European Association of Urology. Accessed: Oct. 16, 2023. [Online]. https://uroweb.org/guidelines/prostate-cancer

[16] Abdul Raheem, R. et al. Can a prostate biopsy be safely deferred on PI-RADS 1,2 or 3 lesions seen on pre-biopsy mp-MRI?. *Arab. J. Urol.* **21**(1), 10–17. 10.1080/2090598X.2022.2119711 (2023). 36818375 10.1080/2090598X.2022.2119711 PMC9930831

[17] Reijnen, J. S. et al. Results from a PI-RADS-based MRI-directed diagnostic pathway for biopsy-naive patients in a non-university hospital. *Abdom. Radiol. N. Y.* **46**(12), 5639–5646. 10.1007/s00261-021-03249-8 (2021). 10.1007/s00261-021-03249-8 PMC8590681 34417637

[18] “MATLAB.” Accessed: Nov. 19, 2024. [Online]. https://www.mathworks.com/products/matlab.html

[19] Holland, D., Kuperman, J. M. & Dale, A. M. Efficient correction of inhomogeneous static magnetic field-induced distortion in Echo Planar Imaging. *NeuroImage* **50**(1), 175–183. 10.1016/j.neuroimage.2009.11.044 (2010). 19944768 10.1016/j.neuroimage.2009.11.044 PMC2819607

[20] Paquin, D., Levy, D., Schreibmann, E. & Xing, L. Multiscale image registration. *Math. Biosci. Eng.* **3**(2), 389–418 (2006). 20361831 10.3934/mbe.2006.3.389

[21] MIM Software Inc. | Precision Care Simplified. Accessed: Nov. 17, 2024. [Online]. http://mimsoftware-5300642.hs-sites.com

[22] Y. Song *et al.* Precise prostate contours: Setting the bar and meticulously evaluating AI performance. 10.1101/2024.10.21.24315771 (2024).

[23] “OnQ^TM^ Prostate - Cortechs.ai.” Accessed: Nov. 14, 2024. [Online]. https://www.cortechs.ai/solution/onq-prostate/

[24] Zhong, A. Y. et al. Automated patient-level prostate cancer detection with quantitative diffusion magnetic resonance imaging. *Eur. Urol. Open Sci.* **47**, 20–28. 10.1016/j.euros.2022.11.009 (2023). 36601040 10.1016/j.euros.2022.11.009 PMC9806706

[25] Conlin, C. C. et al. Background prostate tissue is quantitatively abnormal on MRI in patients with clinically significant prostate cancer. *Radiol. Imaging*10.1101/2022.10.12.22280855 (2022).

[26] Lui, A. J. et al. ReIGNITE RT boost: An international study testing the accuracy and feasibility of using restriction spectrum imaging (RSI) MRI to guide intraprostatic tumor target volume for radiotherapy boost. *Oncology*10.1101/2022.12.13.22283420 (2022).

[27] Kallis, K. et al. Comparison of synthesized and acquired high b-value diffusion-weighted MRI for detection of prostate cancer. *Radiol. Imaging*10.1101/2023.02.17.23286100 (2023). 10.1186/s40644-024-00723-6 PMC11229343 38972972

[28] White, N. S. & Dale, A. M. Distinct effects of nuclear volume fraction and cell diameter on high b-value diffusion MRI contrast in tumors: Diffusion in Tumor Cells. *Magn. Reson. Med.* **72**(5), 1435–1443. 10.1002/mrm.25039 (2014). 24357182 10.1002/mrm.25039 PMC5400014

[29] Sadinski, M. et al. Pilot study of the use of hybrid multidimensional T2-weighted imaging–DWI for the diagnosis of prostate cancer and evaluation of Gleason score. *Am. J. Roentgenol.* **207**(3), 592–598. 10.2214/AJR.15.15626 (2016). 27352026 10.2214/AJR.15.15626 PMC5074540

[30] Wang, S. et al. Hybrid multidimensional T2 and diffusion-weighted MRI for prostate cancer detection. *J. Magn. Reson. Imaging JMRI* **39**(4), 781. 10.1002/jmri.24212 (2013). 23908146 10.1002/jmri.24212 PMC4251798

[31] Sabouri, S. et al. Luminal water imaging: A new MR imaging T2 mapping technique for prostate cancer diagnosis. *Radiology* **284**(2), 451–459. 10.1148/radiol.2017161687 (2017). 28394754 10.1148/radiol.2017161687 PMC5522021

[32] Hepp, T. et al. T2 mapping for the characterization of prostate lesions. *World J. Urol.* **40**(6), 1455. 10.1007/s00345-022-03991-8 (2022). 35357510 10.1007/s00345-022-03991-8 PMC9166840

[33] Chatterjee, A., Harmath, C. & Oto, A. New prostate MRI techniques and sequences. *Abdom. Radiol.* **45**(12), 4052–4062. 10.1007/s00261-020-02504-8 (2020). 10.1007/s00261-020-02504-8 32248259

[34] Lee, G. H. et al. Comparing radiologist performance in diagnosing clinically significant prostate cancer with multiparametric versus hybrid multidimensional MRI. *Radiology* **305**(2), 399–407. 10.1148/radiol.211895 (2022). 35880981 10.1148/radiol.211895 PMC9619199

[35] Chatterjee, A. et al. Changes in epithelium, stroma, and lumen space correlate more strongly with Gleason pattern and are stronger predictors of prostate ADC changes than cellularity metrics. *Radiology* **277**(3), 751–762. 10.1148/radiol.2015142414 (2015). 26110669 10.1148/radiol.2015142414

[36] Liss, M. A. et al. MRI-derived restriction spectrum imaging cellularity index is associated with high grade prostate cancer on radical prostatectomy specimens. *Front. Oncol.*10.3389/fonc.2015.00030 (2015). 25741473 10.3389/fonc.2015.00030 PMC4330697

[37] M. T. Baxter *et al.* 2024 Advanced Restriction imaging and reconstruction Technology for Prostate MRI (ART-Pro): Study protocol for a multicenter, multinational trial evaluating biparametric MRI and advanced, quantitative diffusion MRI for detection of prostate cancer. *medRxiv*10.1101/2024.08.29.24311575 (2024). 10.1016/j.euros.2024.12.003 PMC11730575 39811103

